# HSF4/COIL complex‐dependent R‐loop mediates ultraviolet‐induced inflammatory skin injury

**DOI:** 10.1002/ctm2.1336

**Published:** 2023-07-17

**Authors:** Yi‐qian Feng, Heng Zhang, Jing‐xia Han, Bi‐jia Cui, Lu‐ning Qin, Lei Zhang, Qing‐qing Li, Xin‐ying Wu, Nan‐nan Xiao, Yan Zhang, Ting‐ting Lin, Hui‐juan Liu, Tao Sun

**Affiliations:** ^1^ State Key Laboratory of Medicinal Chemical Biology and College of Pharmacy Nankai University Tianjin China; ^2^ Tianjin Key Laboratory of Early Druggability Evaluation of Innovative Drugs and Tianjin Key Laboratory of Molecular Drug Research Tianjin International Joint Academy of Biomedicine Tianjin China; ^3^ Medical Plastic and Cosmetic Centre Tianjin Branch of National Clinical Research Center for Ocular Disease Tianjin Medical University Eye Hospital Tianjin China

**Keywords:** DNA damage, R‐loop, small‐molecule drugs, transcription factor regulation, UV irradiation

## Abstract

Intense ultraviolet (UV) exposure can cause phototoxic reactions, such as skin inflammation, resulting in injury. UV is a direct cause of DNA damage, but the mechanisms underlying transcriptional regulation within cells after DNA damage are unclear. The bioinformatics analysis of transcriptome sequencing data from UV‐irradiated and non‐UV‐irradiated skin showed that transcription‐related proteins, such as HSF4 and COIL, mediate cellular response to UV irradiation. HSF4 and COIL can form a complex under UV irradiation, and the preference for binding target genes changed because of the presence of a large number of R‐loops in cells under UV irradiation and the ability of COIL to recognize R‐loops. The regulation of target genes was altered by the HSF4–COIL complex, and the expression of inflammation and ageing‐related genes, such as *Atg7*, *Tfpi*, and *Lims1*, was enhanced. A drug screen was performed for the recognition sites of COIL and R‐loop. N6‐(2‐hydroxyethyl)‐adenosine can competitively bind COIL and inhibit the binding of COIL to the R‐loop. Thus, the activation of downstream inflammation‐related genes and inflammatory skin injury was inhibited.

## Graphical abstract

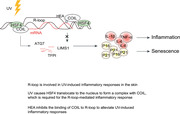

## 1 INTRODUCTION

The skin is the largest organ of the human body^1^ and the first line of defence against external aggression; the skin is essential for maintaining the vital functions of host physiology. A moderate ultraviolet (UV) level can strengthen the body's resistance, whereas strong UV light can lead to the development of phototoxic reactions and inflammation in patients.^2^ Skin injured by UV radiation (UVR) shows edematous erythema or even oedema, forming blisters; the patient will feel an itching, burning or tingling sensation. The skin contains an intricate network of immune cells in tissues; these cells are crucial for host defence and tissue homeostasis^3^ and block many external threats. The study of factors affecting skin health is gradually gaining attention in recent years. A poor diet can lead to a weak skin barrier^4^ and the development of melanoma.^5^ Some external conditions can induce various skin diseases of varying complexity and diversity, including redox‐dependent pigmentation^6^ and allergic diseases, infectious diseases, atrophy, pigmentation changes and even skin cancer.^7^ UVR from sunlight is traditionally known to mutagenize DNA and damages proteins and lipids, causing deleterious effects. It is considered a tumour initiator and promoter.

The damaging effects of UV on the skin are caused by direct cell injury and indirect changes in immune function. Excessive UV exposure can damage collagen, elastin and glycosaminoglycans, leading to connective tissue injury in the skin and thereby affecting the skin's strength, elasticity and hydration.^8^ UV is epidemiologically associated with the three most common skin cancers, namely, basal cell carcinoma, squamous cell carcinoma^9^ and malignant melanoma.^10^ The aetiology of basal cell carcinoma has been confirmed; when UVR is applied to adjoining pyrimidines, covalent bonds are formed, causing the formation of mutagenic photoproducts, such as cyclopyrimidine dimers (TT) and pyrimidine‐pyrimidine (6‐4) adducts.^11^ This suggests that UVR leads to direct UV signature mutations.^12^ UVR can indirectly damage DNA and RNA by forming reactive oxygen intermediates.^13^ UVR may trigger autoimmunity through a stimulator of interferon gene‐dependent innate immune signalling,^14^ inducting type I interferons that exert effect against pathogens.^15^, ^16^ Mice exposed to high doses of UVR are significantly impaired in their ability to present antigens in the spleen.^17^ This systemic effect is largely due to the UVR‐induced release of keratinocyte‐derived soluble mediators.^18^ Studies on UV‐induced skin inflammatory damage have confirmed that some inflammatory cytokines play an important role in the complex response to UV. These inflammatory cytokines can generate inflammation‐related immune responses by altering the expression of adhesion molecules and cytokines of resident cell populations and by inducing the migration of nonresident cells to this area.^18^ UV‐ and transcription factor MITF‐related skin pigmentation mechanisms are elucidated in genetic and epigenetic studies on skin tone,^19^ whereas the key transcription factors and related mechanisms initiated by UV‐induced skin cells in response to UV‐stimulated damage are still unclear.

In this study, starting from the transcriptome sequencing data of sun‐exposed or unexposed skin tissues, bioinformatics was used to identify key transcription factors that induce this process. Cell biology and biochemistry experiments were performed to determine whether the transcription factors HSF4 and COIL form a novel complex under UV stimulation. The important role of the HSF4–COIL complex was investigated. By inducing the massive production of an R‐loop,^20^ a high‐level nucleic acid structure trans‐regulated the transcription of many genes related to inflammation and senescence.

## 2 RESULTS

### 2.1 HSF4 is a key transcription factor regulating UV‐induced inflammation and forms an HSF4–COIL complex with the coil protein COIL under UV induction

**FIGURE 1:**
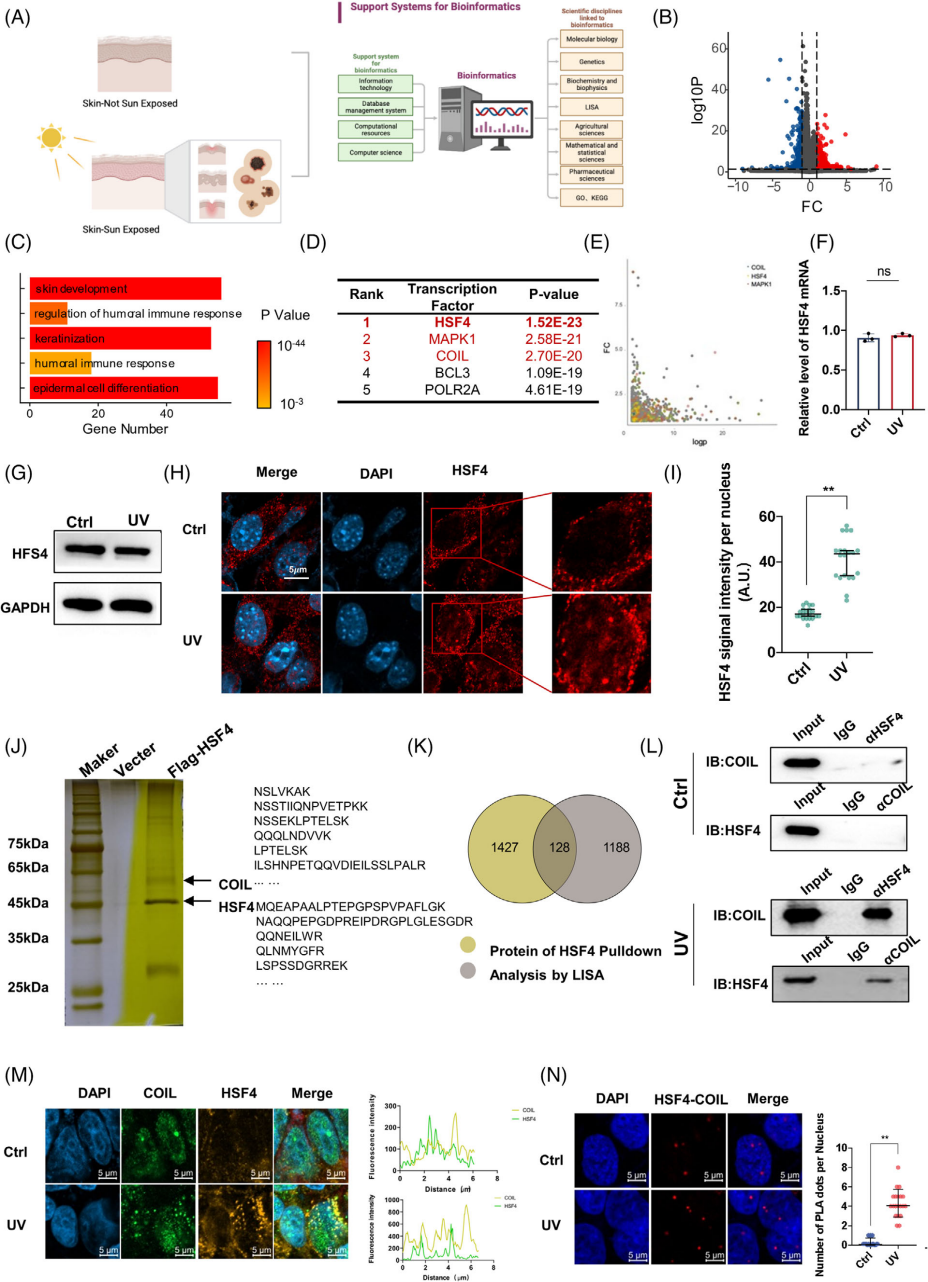
HSF4 is a key transcription factor regulating ultraviolet (UV)‐induced inflammation and forms an HSF4–COIL complex with COIL under UV induction. (A) Strategies for studying human skin databases. (B) Enrichment analysis of differentially expressed genes induced by ultraviolet radiation showed that 340 genes were upregulated (red) and 500 genes were downregulated (blue). (C) Pathway enrichment analysis of genes whose expression was upregulated after UV induction showed that a large number of genes were enriched in immune‐inflammation‐related pathways after UV induction. (D) The transcription factors that mainly regulate the upregulation of gene expression were analyzed by landscape in silico deletion analysis (LISA). (E) The downstream genes regulated by the top three transcription factors were marked by bioinformatics. (F) The mRNA expression of intracellular HSF4 in the experimental group and UV‐treated group. (G) Detection of HSF4 protein expression levels in cells before and after UV induction by Western blot. (H) The location and expression of HSF4 in the cells of the control group and UV‐treated group were detected by immunofluorescence, and the intensity of the HSF4 fluorescence signal in the nucleus of the control group and UV‐treated group was quantified. (I) Quantification of HSF4 fluorescence signal intensity in nuclei from control and UV‐treated groups. (J) GST‐pulldown assay with HSF4 protein in NIH‐3T3 cells after UV stimulation. The peptides were identified in the same way as above with this combined sequence database. (K) Venn diagram of HSF4 pull‐down and LISA analysis results. (L) Co‐immunoprecipitation (Co‐IP) assay protein–protein interactions were analyzed by Co‐IP experiments. The interaction test of HSF4 and COIL was performed on NIH‐3T3 cells before and after UV induction. (M) Immunofluorescence (IF) analysis of the colocalization of HSF4 and COIL before and after UV induction: HSF4, green; COIL, yellow. (N) The performed proximity ligation assay (PLA) experiments demonstrated enhanced HSF4‐COIL intranuclear interaction after UV induction. Error bars represent mean  ±  s.d.; *p ≤ 0.05; **p ≤ .01; ***p ≤ .0005.

To investigate the mechanisms of inflammatory skin damage and ageing caused by sun exposure, we downloaded transcriptome sequencing data of skin tissues from the Genotype‐Tissue Expression Project database from UCSC Xena and imported them into R for analysis (Figure 1A). Sun‐exposed and unexposed skin samples were screened separately and divided into two groups, and difference analysis was performed with the DEseq2 package. A total of 840 genes (Table S3) with |log2FC| > 1 and P < 0.05 were obtained; 340 of these genes were significantly upregulated in the sun‐exposed group compared with the unexposed group (Figure 1B). The results of the KEGG pathway enrichment analysis showed that 193 genes were associated with various inflammatory pathways (Figure 1C), and a large number of genes were enriched in pathways related to skin development and immune response, such as epidermal cell differentiation, keratinization and skin development. This result coincided with the fact that sun‐exposed skin tissues are more prone to inflammatory responses, such as melanosis. Epigenetic landscape in silico deletion analysis (LISA) integrates H3K27ac chromatin immunoprecipitation‐sequence (ChIP‐seq) and Dnase‐seq with transcriptional regulator ChIP‐seq or imputed transcriptional regulator binding sites to predict transcriptional regulators that regulate a query gene set.^21^ To study the transcriptional regulatory proteins that play a major role in the skin under sunlight or UV irradiation, the LISA algorithm was used to find the main contributing transcriptional regulatory proteins that regulate these genes. Proteins, such as HSF4, MAPK1, COIL and BCL3, all had a certain credibility, and HSF4 showed the highest credibility (Figure 1D) and may thus have played a leading role. HSF4 is a key transcription factor in cellular responses to physical stimuli, such as heat.^22^ Based on the analysis of the ChIP‐seq public database (http://cistrome.org/db/; CistromeDB: 38644), HSF4 can regulate 17.14% of the upregulated genes in Figure 1B (Figure 1E). To investigate the behaviour of HSF4 under UVR, we used an NIH‐3T3 cell line,^23^ which is widely used in skin studies, to establish a UV‐induced model. NIH‐3T3 is also used in skin phototoxicity tests. No significant changes were found in the mRNA expression (Figure 1F) and protein levels (Figure 1G) of HSF4 under UV stimulation. Further cellular immunofluorescence (IF) experiments showed that the HSF4 signal was significantly elevated in the nucleus after UV irradiation, suggesting that its entry into the nucleus under UV action plays an important regulatory function (Figures 1H,I).

Next, we sought to determine whether HSF4‐mediated transcription upon UV irradiation involves the recruitment of other transcriptional cofactors. After the UV treatment of NIH‐3T3 cells, pull‐down experiments were performed with HSF4 (Figure 1J), and the proteins obtained by the pull‐down experiments were identified by mass spectrometry. The proteins identified to interact with HSF4 intersected with the proteins obtained from LISA analysis in Figure 1D (Figure 1K). COIL (coilin) interacted with HSF4 and ranked third in the LISA analysis. HSF4–COIL may form conformer structures in response to UV stimulation, and this complex is a key regulatory structure for UV‐induced inflammatory responses. Therefore, HSF4–COIL interactions in cells under UV stimulation were verified through co‐immunoprecipitation (Co‐IP) experiments. Under normal conditions, HSF4 had no significant interaction with COIL (Figure 1L). IF experiment showed the low intracellular co‐localization of HSF4 and COIL without UV treatment, but the co‐localization signal increased after UV induction. The proximity ligation assay (PLA) experiment showed that the HSF4–COIL interaction signal significantly increased after UV induction (Figure 1M), indicating that UV induced the interaction of HSF4 and COIL to form a complex (Figure 1N).

### 2.2 HSF4–COIL complex activates the transcription and expression of genes related to inflammation and senescence

**FIGURE 2:**
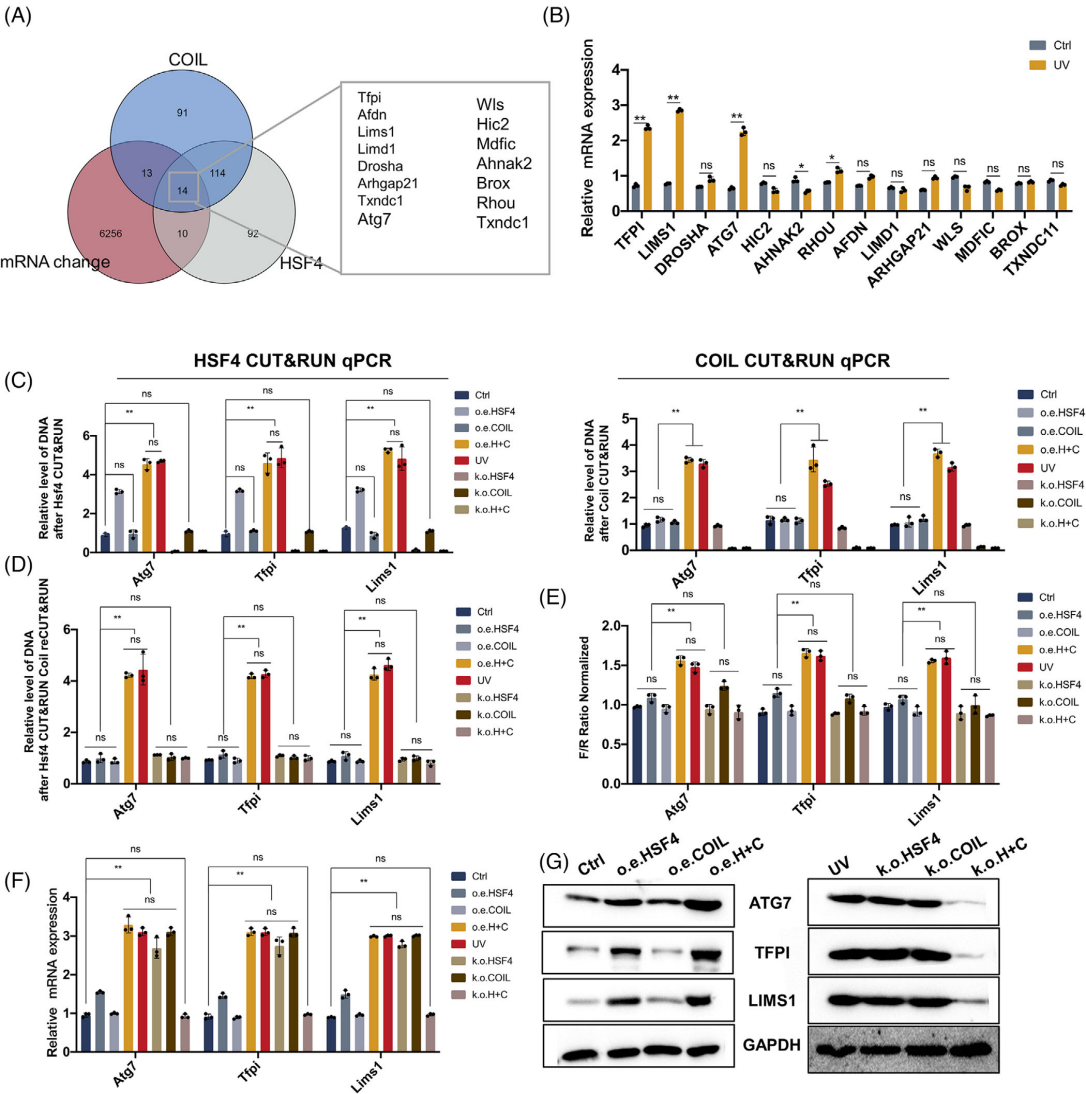
HSF4–COIL complex activates the transcription and expression of genes related to inflammation and senescence (A) The downstream genes regulated by COIL and HSF4 after ultraviolet (UV) stimulation and the gene Venn with differential messenger RNA (mRNA) expression under UV stimulation showed all coregulated genes. (B) Quantitative polymerase chain reaction (qPCR) revealed differences in the expression of co‐regulated genes after UV stimulation. (C) Chromatin immunoprecipitation (ChIP)–qPCR analysis of HSF4 and COIL mRNA expression levels of Atg7, Tfpi and Lims1 before and after UV induction. (D) ChIP–reChIP verified the co‐regulation of HSF4 and COIL on Atg7, Tfpi and Lims1. (E) The luciferase reporter assay detected that the transcription factor HSF4 enhanced the transcriptional activation of Atg7, Tfpi and Lims1 before and after UV treatment. (F) Differences in Atg7, Tfpi and Lims1 mRNA expression before and after UV induction. (G) Differential expression of Atg7, Tfpi and Lims1 protein before and after UV induction. Error bars represent mean  ±  s.d.; *p ≤ 0.05; **p ≤ .01; ***p ≤ .0005.

COIL is detected at the site of DNA damage.^24^ However, although the aggregation of COIL is associated with UV induction,^25^, ^26^ the function of COIL in UV‐induced DNA damage is unclear. To further study the expression of genes regulated by the interaction between HSF4 and COIL, on the one hand, we conducted CUT&RUN on HSF4 and COIL on cells treated with UV to determine which genes were regulated by each transcription factor. On the other hand, we performed transcriptome analysis (mRNA sequencing) on NIH‐3T3 treated with UV (Table S4). The sequencing data showed that the mRNA of 1716 genes changed significantly after UV induction compared with the control group (the standard was |log2FC| > 1 and *p* < 0.05). Figure 2A shows HSF4 CUT&RUN, COIL CUT&RUN and upregulated genes after UV induction. We thought that the intersection of the three groups of genes is significantly regulated by the interaction of HSF4 and COIL, with a total of 14 genes. Multiple genes in the intersection were associated with inflammation wound production, and cellular senescence (Figure 2B). *Atg7*, *Lims1* and *Tfpi* genes, which had the most significant differences in expression under UV stimulation, were selected for further study. Atg7,^27^ Lims1^28^ and Tfpi^29^ have functions in the regulation of inflammation and cell senescence. We attempted to determine whether the manipulation of HSF4 or COIL expression is sufficient to produce a change similar to that caused by UV irradiation. Interestingly, we found that the overexpression or knockdown of COIL is insufficient to alter the expression of Atg7, Tfpi and Lims1. Furthermore, the knockdown of HSF4 had no effect on the expression of inflammatory or age‐related genes, and its overexpression had only a slight effect (Figure 2C). However, the simultaneous knockdown or overexpression of HSF4 and COIL resulted in changes in Atf7, Tfpi and Lims, suggesting that the two transcription factors can alter gene expression in response to UV exposure. We suggested that in the HSF4–COIL complex, HSF4 may play a major role in the regulation of downstream genes; this regulatory function depends on the appearance of COIL proteins (Figure 2C). In this regard, the role of COIL proteins in the HSF4–COIL complex remains to be investigated. To verify whether HSF4 and COIL coregulate downstream inflammatory and senescence genes, we performed ChIP–reChIP experiments. The ChIP–reChIP–quantitative polymerase chain reaction (qPCR) data demonstrated that HSF4 and COIL coregulate downstream inflammatory and senescence genes (Figure 2D). Dual‐luciferase assays were performed to validate the results (Figure 2E). The mRNA and protein expression levels of the target genes were detected before and after UV irradiation injury (Figures 2F,G). The expression of downstream Atg7, Tfpi and Lims1 was significantly upregulated after UV stimulation, and the combined knockdown of HSF4 and COIL alleviated the pathological upregulation of downstream genes.

All these results suggested that UV promoted the formation of an HSF4–COIL complex and promoted the expression of inflammatory and senescence genes.

### 2.3 R‐loop was involved in UV response mediated by HSF4–COIL

**FIGURE 3:**
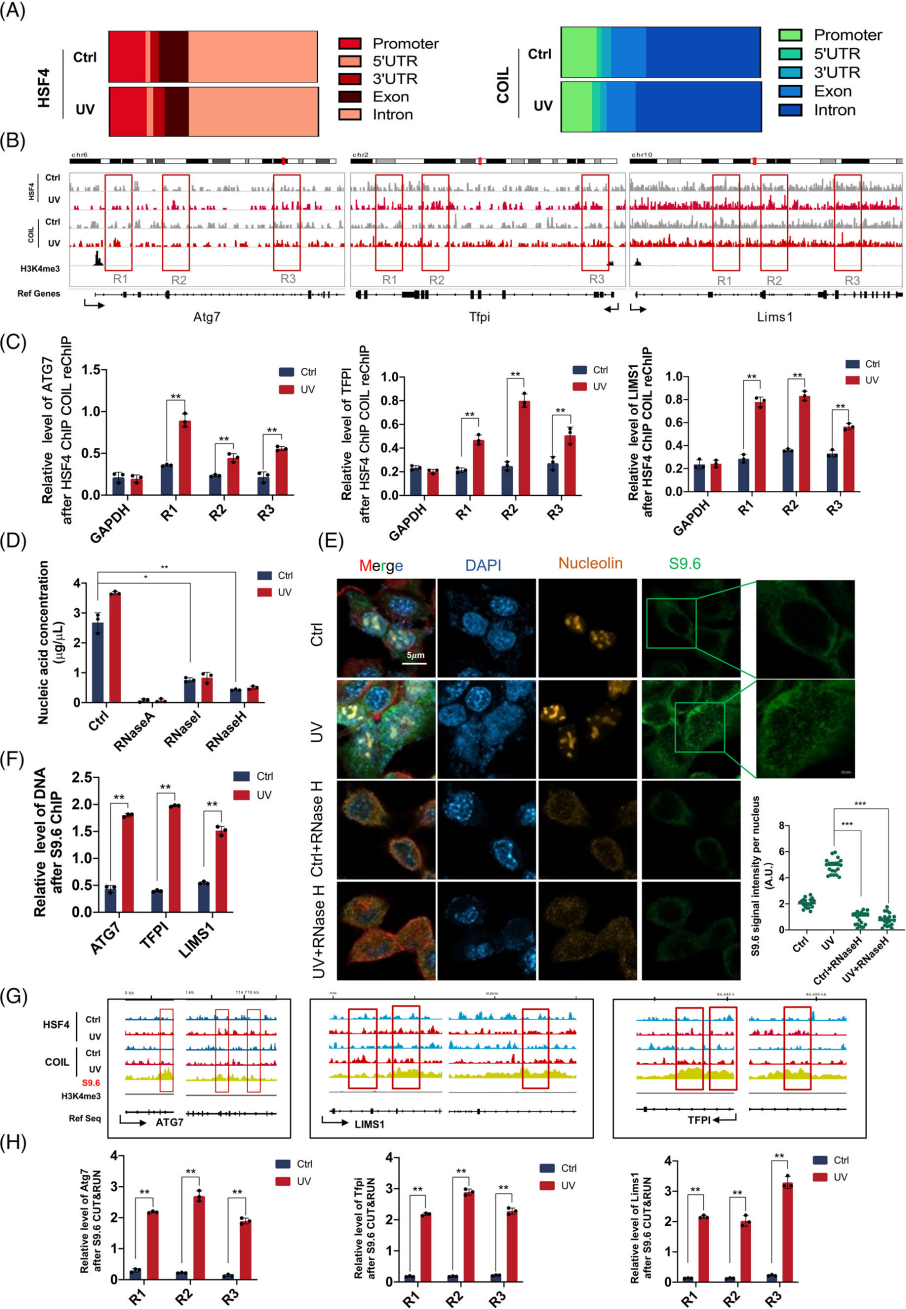
R‐loop is involved in ultraviolet (UV) response mediated by HSF4–COIL. (A) The distribution statistics of HSF4 and COIL UV‐induced/non‐UV‐induced CUT&RUN‐seq binding peak positions. (B) CUT&RUN ‐seq showed enhanced binding of HSF4 and COIL to Atg7, Tfpi and Lims1 after UV induction. (C) Chromatin immunoprecipitation (ChIP)‐reChIP showed enhanced binding of Atg7, Tfpi and Lims1 to HSF4 and COIL in the promoter regions (R1, R2 and R3) after UV induction. (D) RNA content of HSF4, COIL and ChIP samples treated with different nucleases. (E) The immunofluorescence image shows that the content of the R‐loop increases after UV stimulation, and RNaseH can specifically degrade the R‐loop. Statistical charts make statistics on the fluorescence signal of the R‐loop. (F) DRIP‐qPCR showed that R‐loop enhanced the regulation of Atg7, Tfpi and Lims1 after UV induction. (G) CUT&RUN‐seq showed the colocalization of Atg7, Tfpi, Lims1 and R‐loop peaks under UV induction. (H) DRIP‐qPCR showed that R‐loop mediates the expression of Atg7, Tfpi and Lims1 after UV induction.Error bars represent mean  ±  s.d.; *p ≤ .05; **p ≤ .01; ***p ≤ .0005.

The CUT&RUN of HSF4 and COIL in the two groups of cells was performed after Ctrl and UV treatments, and the obtained CUT&RUN data was analyzed. The proportion of HSF4‐bound promoter region increased after UV treatment (Figure 3A), whereas the proportion of COIL‐bound exon region decreased significantly. The binding peaks of HSF4 and COIL at the three genes (*Atg7, Tfpi* and *Lims1*) showed that UV induction shifted the binding gene positions of HSF4 and COIL (Figure 3B). Then, ChIP–reChIP experiments were performed for verification. *Atg7, Tfpi* and *Lims1* were confirmed to be coregulated by HSF4 and COIL, and the expression levels of the genes were affected by changes in target gene binding sites. Moreover, we found the specific peak of the CUT&RUN sequencing data in the R1, R2 and R3 regions. This specific peak was evaluated by the presence of a peak that did not appear in the control group after UV irradiation, and we considered this peak to be UV specific. The position and sequence of the DNA corresponding to this peak were found by IGV software (provided in Table S2), and the PCR primers were designed using this DNA sequence as a template (Figure 3C). Given the shift in the position of HSF4 and COIL‐binding genes under UV stimulation, we believed that the transcription regulation behaviour of HSF4 and COIL changed under UV stimulation (Figure 3B). R‐loop‐mediated gene expression enhancement had been demonstrated in previous studies.^30^ This type of transcriptional activation can regulate the chromatin structure around a promoter, leading to the upregulation of gene expression. Based on the shift in the position of HSF4 and COIL‐binding genes under UV stimulation, this phenomenon is likely due to the involvement of the R‐loop in transcriptional regulation.

After RNA quantification of the ChIP‐captured DNA fragments, a large amount of RNA was indeed present, and RNA content in the UV‐treated group was significantly higher than that in the control group. The type of RNA involved was further investigated. Three RNA enzymes (RNaseA, RNaseI and RNaseH^20^) were used for the treatment. The RNA concentration decreased after RNaseH and RNaseI treatments, and no RNA was found after RNaseA treatment. The degradation of RNA by RNaseH in the DNA–RNA heteroduplexes confirmed that the R‐loop is involved in transcriptional regulation (Figure 3D). Therefore, the R‐loop, a nucleic acid high‐level structure, may be involved in UV‐induced inflammatory injury. R‐loop regulates gene expression and chromatin structure and is a result of genomic instability.^31^ An HSF4–COIL complex may be bound to the R‐loop, mediating the transcription of inflammatory factors, such as IL‐1β, IL‐6 and TNF‐α, and accelerating skin injury.

The anti‐DNA–RNA hybrid clone S9.6 is a highly specific mouse monoclonal antibody that targets DNA–RNA hybrids and has been tested in affinity binding assay, ChIP, ChIP‐seq, Dot blot and IP.^31^ The R‐loop content of NIH‐3T3 cells after UV‐induced significantly increased, whereas that of cells treated with RNaseH significantly decreased (Figure 3E). DNA–RNA immunoprecipitation (DRIP) using an S9.6 antibody was conducted on both cell groups to detect the R‐loop levels in three downstream genes by qPCR. The binding of R‐loops in the downstream genes *Atg7*, *Tfpi* and *Lims1* was significantly enhanced under UV stimulation (Figure 3F). Based on the peaks of S9.6 DRIP‐seq and CUT&RUN, the ORF regions of *Atg7*, *Tfpi* and *Lims1* showed a large number of R‐loops after UV stimulation (Figures 3G,H). R‐loop was involved in the regulation of UV response by HSF4–COIL.

### 2.4 UV‐induced R‐loop pathological increase is dependent on the HSF4–COIL complex

**FIGURE 4:**
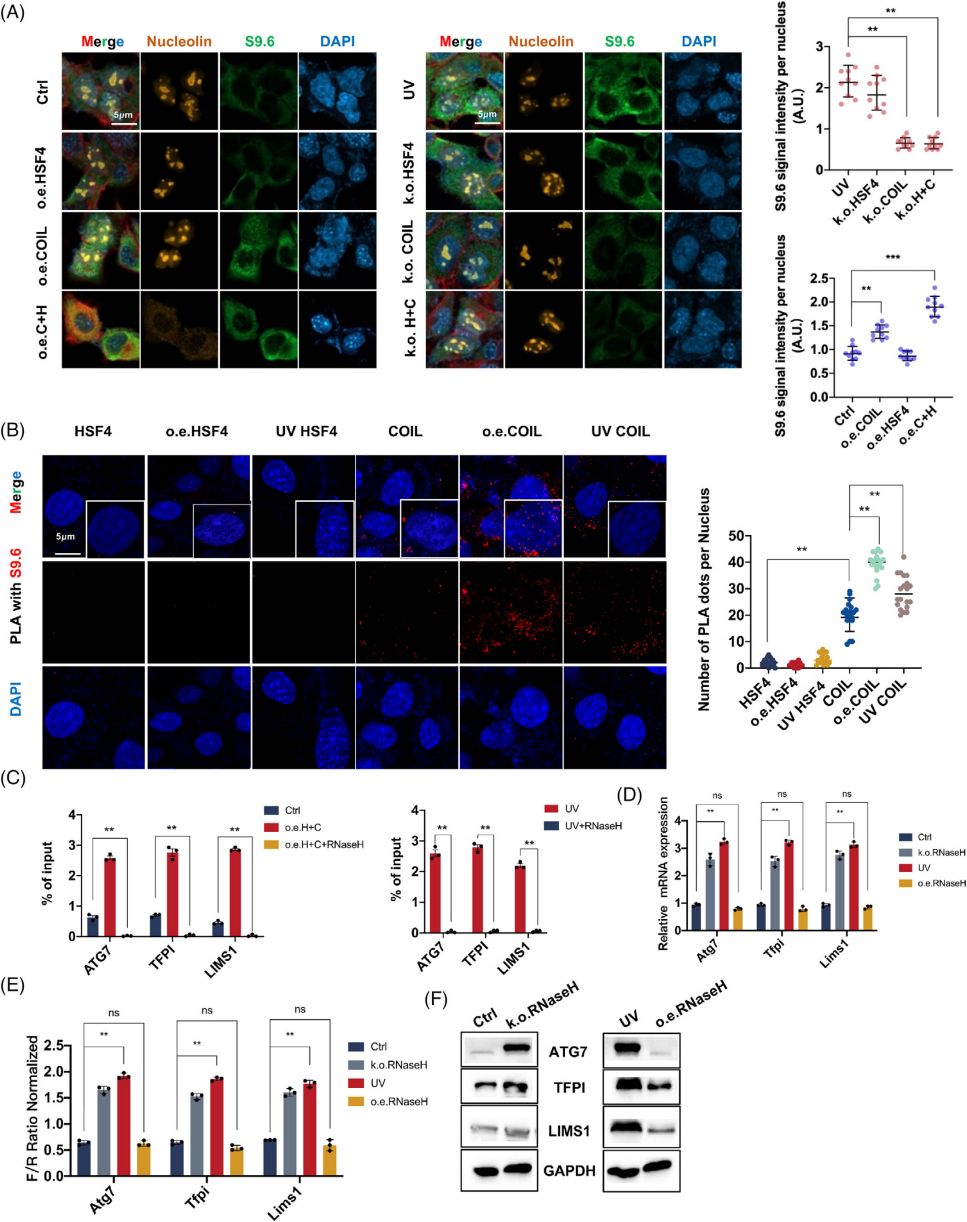
Ultraviolet (UV)‐induced R‐loop pathological increase is dependent on the HSF4–COIL complex. (A) Immunofluorescence showing the effects of overexpression/knockout HSF4 and COIL on R‐loop expression before and after UV stimulation. Statistical graphs are used to perform statistics on the R‐loop fluorescence signal. (B) Proximity ligation assay (PLA) experiments showed colocalization analysis of R‐loops after the overexpression/knockdown of HSF4 and COIL, proving that COIL is a key protein that binds to R‐loops. Statistical plots count the fluorescent signals generated by colocalization. (C) DRIP–qPCR demonstrated the regulation of Atg7, Tfpi and Lims1 by R‐loops. (D) The luciferase reporter gene showed the transcriptional activities of Atg7, Tfpi and Lims1 under different R‐loop conditions. (E, F) Messenger RNA (mRNA) and protein expression levels of Atg7, Tfpi and Lims1 under different R‐loop conditions. Error bars represent mean  ±  s.d.; *p ≤ .05; **p ≤ .01; ***p ≤ .0005.

To further investigate what factors contribute to the pathological increase in R‐loops and how it is involved in the inflammatory response, we knocked out or overexpressed HSF4 and COIL in both groups. The results of the IF experiments showed that the overexpression of HSF4 under control conditions did not contribute to inducing an increase in the R‐loop, and no significant difference was found in R‐loop expression after the overexpression of COIL. The results of this experiment were further confirmed in UV‐stimulated cells. The high expression of the R‐loop after UV stimulation significantly reduced the R‐loop expression by the co‐knockout COIL and HSF4 (Figure 4A). The HSF4–COIL complex bound the R‐loop to prevent its degradation by RNaseH. The binding of the R‐loop to the complex enhanced the regulation of inflammatory and senescence genes by the transcription factor HSF4.

The colocalization phenomenon of HSF4, COIL and R‐loop was verified through PLA experiments. The role of HSF4 and R‐loop was significantly weaker. COIL and R‐loop had a rather strong colocalization phenomenon (Figure 4B). Therefore, COIL played the main role in binding the R‐loop in the HSF4–COIL complex. To reconfirm whether the long‐term existence of R‐loop was related to the HSF4–COIL complex, HSF4 and COIL overexpression under normal conditions was verified through DRIP‐qPCR, and the results showed that the expression levels of downstream inflammation and ageing‐related genes increased. Meanwhile, after RNaseH treatment, the expression of inflammatory and ageing‐related genes, such as *Atg7*, *Tfpi* and *Lims1*, significantly decreased, and the expression almost did not increase compared with input (Figure 4C). A luciferase reporter system was designed for verification (Figure 4D). After the high expression of knockout RNaseH on the R‐loop, the transcriptional activity of this position significantly increased, and UV stimulation reversed the effect of knocking out RNaseH. Western blot and qPCR experiments (Figures 4E,F) showed that the mRNA and protein content of *Atg7, Tfpi* and *Lims1* significantly increased after the overexpression of the R‐loop, and the effects of R‐loop were reverted after UV induction and knockout of RNaseH.

These results showed that the HSF4–COIL complex had a certain structure that bind to the R‐loop produced in large quantities under UV stimulation, and the stability of the R‐loop was maintained. After the HSF4–COIL complex bound to the R‐loop, it protected the R‐loop from intracellular self‐regulated degradation, particularly that caused by RNaseH. Furthermore, R‐loop binding was a key factor that led to the altered promoter binding site of HSF4 and COIL. This alteration resulted in the enhanced regulation of the HSF4–COIL complex on the downstream inflammation and senescence‐related genes, such as *Atg7*, *Tfpi* and *Lims1*.

### 2.5 HSF4–COIL complex and R‐Loop promoted skin inflammation and ageing under UV conditions

**FIGURE 5:**
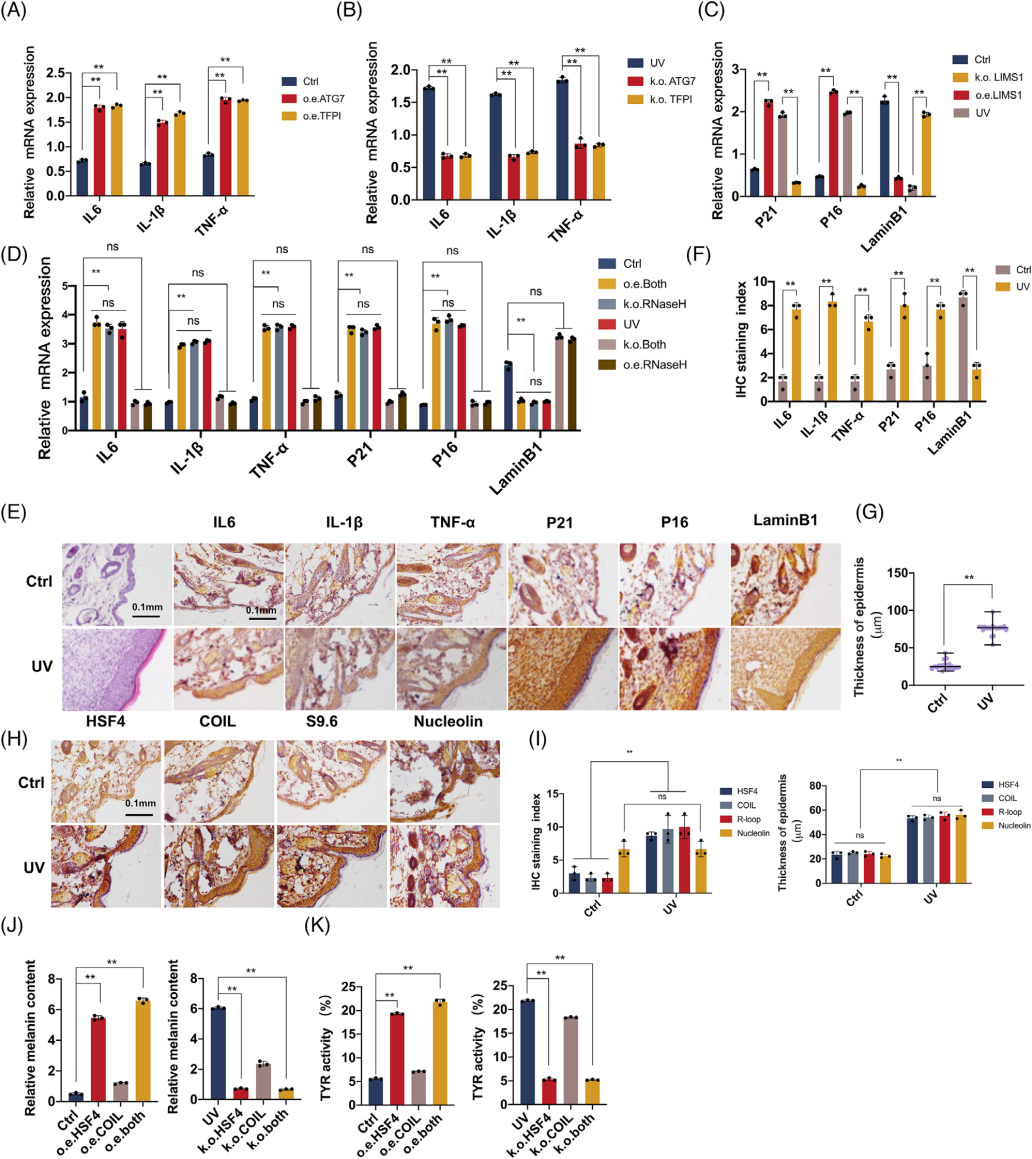
HSF4‐COIL complex and R‐Loop promoted skin inflammation and ageing under UV conditions. (A, B) Effect of knockout or overexpression of Atg7 (A) and Tfpi (B) on the expression of inflammatory factors. (C) Effect of knocking out or overexpressing Lims1 on the expression of age‐related markers. Error bars represent mean  ±  s.d.; *p   < .05; **p   < .01; ***p   <  .0005. (D) qPCR shows the effects of inflammatory factors and senescence factors before and after UV induction, and after simultaneous knockout/overexpression of HSF4, COIL and RNaseH. (E–G) HE staining showed the changes of epidermal thickness before and after UV induction, and immunohistochemistry showed the expression of inflammatory factors and aging factors in the skin after UV induction. (H) IHC showed that the expression of HSF4, COIL, and R‐loop in the tissues after UV induction changed, accompanied by epidermal thickening. (I) The score of immunohistochemistry showed that the expression levels of HSF4, COIL and R‐loop increased after UV induction, and the score of skin thickness proved that UV induction leads to skin thickening. (J) The expression of melanin content before and after UV induction and after knockout/overexpression of HSF4 and COIL. (K) The tyrosinase activity before and after UV induction, and after knockout/overexpression of HSF4 and COIL. Error bars represent mean  ±  s.d.; *p ≤ .05; **p ≤ .01; ***p ≤ .0005.

Atg7, as an autophagy protein, is a key regulator of endothelial cell inflammation and permeability.^32^ When Atg7 was knocked down using siRNA in the study of intestinal inflammation, the expression of proinflammatory molecules IL‐1β, IL‐6 and TNF‐α were significantly attenuated. A high expression of Atg7 can lead to the malignant enhancement of hepatocellular carcinoma. As a key anticoagulant protein of endothelial and platelet species, Tfpi was elevated in malignant diseases,^33^ and a highly pathological coagulation phenomenon triggered an immune response that ultimately led to inflammation.^34^ Lims1, a senescent cell antigen‐like structural domain, was first identified in a cDNA library of human senescent erythrocytes and was closely associated with cellular senescence. Therefore, the relationship between Atg7 and Tfpi and inflammatory factors in NIH‐3T3 was verified, and the relationship between Lims1 and senescence in epithelial cells was verified using the classical cellular senescence markers P21, P16 and LaminB1^35^, ^36^ (Figures 5A–C).

The regulatory role of HSF4 and COIL knockout or overexpression on UV‐induced skin inflammation and ageing was verified (Figure 5D). The overexpression of HSF4 and COIL and the knockdown of RNaseH under normal conditions can lead to inflammation and cellular senescence, and simultaneous knockdown of HSF4 and COIL or overexpression of RNaseH under UV conditions alleviated this inflammation and cellular senescence.

Given that the activation of UV‐induced inflammatory response and ageing‐related genes are more likely regulated by the synergistic effects of HSF4 and COIL rather than either of the transcription factor alone, we evaluated the skin thickness parameters of organoids^37^ after their co‐overexpression or knockout. We also studied the expression of mRNA and proteins related to inflammation and ageing. After UV induction, the cuticle of the skin organoids was significantly thickened, which showed that the metabolism of cells was slowed down and ageing cutin accumulated under UV stimulation. The overexpression of HSF4‐COIL undergoes stratum corneum thickening (Figures S1A,B). We also detected the mRNA and protein levels of inflammatory factors and senescence‐related markers in skin organoids (Figure S1C,D). Under UV stimulation, the expression of inflammatory factors and senescence‐related markers in skin organs increased significantly. This phenomenon was alleviated when the skin organoid model in HSF4–COIL were knocked out, consistent with the conclusions at the molecular and cellular levels. Furthermore, a UV‐induced model for C57BL/6 mice was constructed, and the dorsal hair removal area was treated with UV irradiation for 20 min three times a week. After 1 month, the dorsal skin was collected for H&E and immunohistochemical staining. UV‐induced led to a significant increase in the epidermal thickness of the dorsal surface of the mice, and significant signs of inflammation and ageing were observed (Figures 5E–G). We found that the expression of the R‐loop significantly increased in the skin tissue of the mouse back after UV induction (Figures 5H,I). Meanwhile, the quantitative melanin experiment and tyrosinase activity experiment showed that HSF4 and COIL can regulate UV‐induced inflammation and ageing and UV‐induced melanin deposition (Figures 5J,K).

### 2.6 Nucleotide analogue N6‐(2‐hydroxyethyl)‐adenosine can reduce the presence of R‐loop

The key step in the strong inflammatory response induced by UV irradiation was the prolonged presence of the R‐loop, that is, the binding of the HSF4–COIL complex to the R‐loop. In the above experiments, COIL, as a special coil protein, was confirmed to be the key to binding to the R‐loop. Whether inhibiting the binding of COIL to the R‐loop can protect cells from entering a state of excessive inflammatory response is unclear.

**FIGURE 6:**
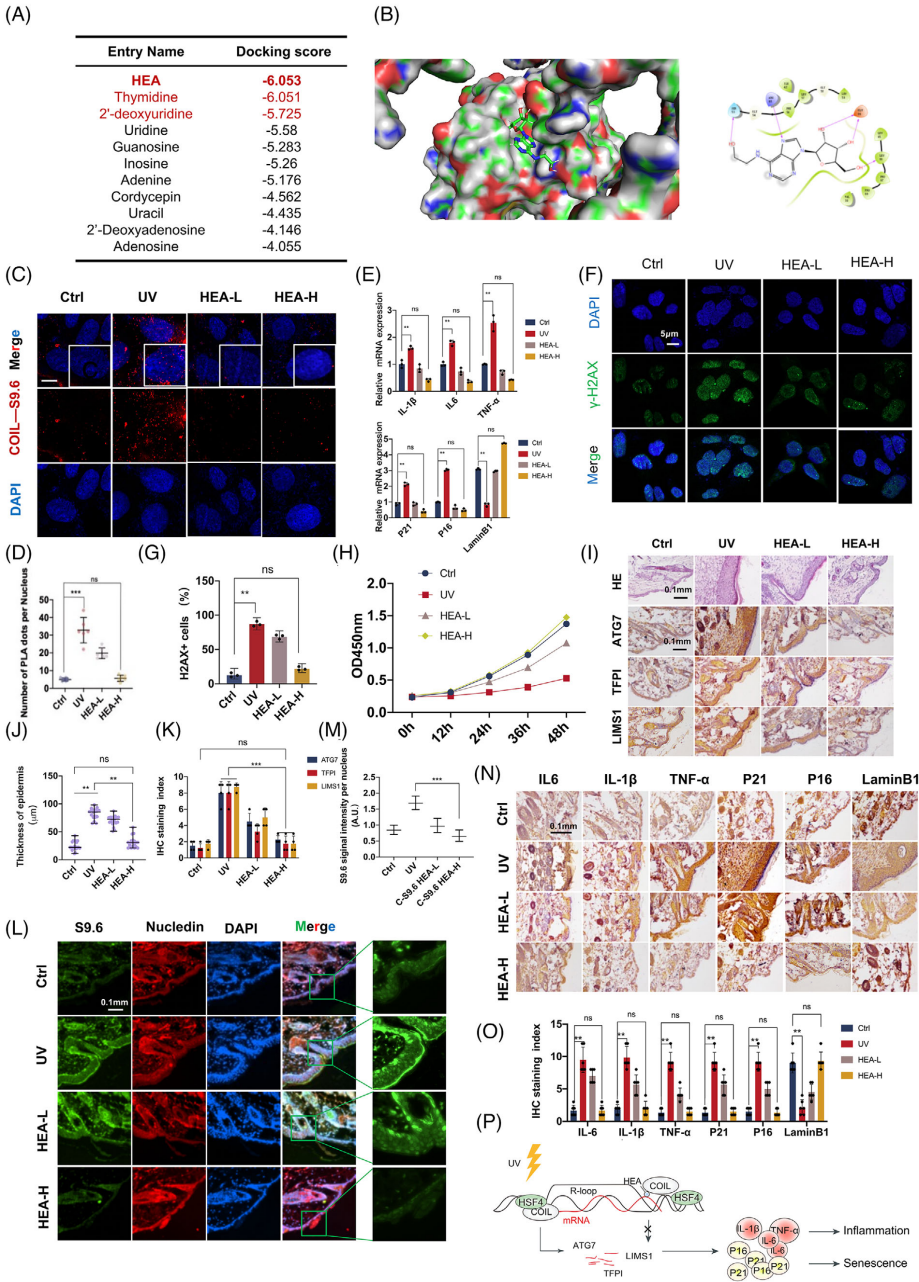
Nucleotide analogue N6‐(2‐hydroxyethyl)‐adenosine (HEA) can reduce the presence of R‐loop. (A) The docking energies predicted by the docking conformation of various nucleoside analogue drugs combined with R‐loop were ranked, and the docking results were evaluated. (B) Structure display of COIL binding to R‐loop, and structure display of COIL binding to N6‐(2‐hydroxyethyl)‐adenosine (HEA). (C, D) Proximity ligation assay (PLA) experiments verified that HEA would attenuate the binding of COIL to the R‐loop, and the statistical graph was the number of co‐localized fluorescent signals in the nucleus. (E) qPCR showed that high and low doses of HEA reduced the expression of downstream inflammatory factors. (F, G) Immunofluorescence test showed that after UV induction γ‐ The increased expression of H2AX indicates an increase in DNA damage. (H) Cck8 showed that the activity of cells treated with HEA‐H/L was significantly higher than that of cells treated with UV. (I–K) Immunohistochemistry of mouse back skin showed that Atg7, Tfpi and Lims1 increased pathologically after UV irradiation, and the expressions of Atg7, Tfpi and Lims1 decreased after smearing HEA on the back of the UV model mice. (L) Immunofluorescent techniques on fixed skin tissue showed that HEA reduced R‐loop expression at the tissue level. (M) Statistics of the skin thickness of the mice after UV irradiation and HEA treatment, the immunohistochemical score (H‐score) was then calculated. And the statistics of the S9.6 fluorescence signal in the immunofluorescence nucleus on the tissue. (N, O) Mouse skin IHC showing the effect of HEA treatment on the expression of inflammatory factors. Statistical graphs are immunohistochemical scores. (P) Proposed model for the role of HSF4‐COIL complex and R‐loop in inflammatory injury caused by UV radiation. Error bars represent mean  ±  s.d.; *p ≤ .05; **p ≤ .01; ***p ≤ .0005.

A strategy was designed to use natural nucleic acid analogues to compete with the R‐loop for binding to COIL and inhibit the binding of COIL to the R‐loop. Molecular docking was performed for five nucleic acid analogues in the active region of the R‐loop binding the COIL protein. The strongest binding (Figure 6A) was N6‐(2‐hydroxyethyl)‐adenosine (HEA). The interface between HEA and COIL was similar to that between COIL and the R‐loop. In Figure 6B, we described the mutual recognition of proteins and small molecules through spatial matching to form molecular complexes and predict the structure of the complexes. By placing the ligand molecule in the active site of a receptor macromolecule and observing the conformation of the small molecule bound to the protein, we predicted that the small molecule binds to the COIL protein by mimicking the nucleic acid structure. As such, the COIL protein binding site became occupied and unable to bind to the R‐loop, thereby protecting the cell from eliciting excessive inflammatory responses. PLA experiments found that HEA can dose‐dependently attenuate the binding of COIL to the R‐loop (Figures 6C,D). After the UV‐induced model was established, high and low doses of HEA were used to treat NIH‐3T3 cells. The mRNA levels of inflammatory factors IL6, IL‐1β and TNF‐α were significantly reduced (Figure 6E). We used the marker of DNA damage γ‐ H2AX to detect the degree of DNA damage to the cells after UV and HEA protection. Under UV irradiation, the level of nuclear γ‐H2AX increased significantly. Under the protection of HEA, the level of nuclear γ‐H2AX was close to that of the control group. Hence, HEA exerted a good effect that protected cells from DNA damage (Figures 6F,G). CCK8 experiment showed that HEA can protect the cell state and make the cell vitality close to the control group (Figure 6H). Animal experiments showed that the back skin of the mice was significantly thickened under UV stimulation (Figures 6I,J), and the expression of related inflammation and ageing genes *Atg7*, *Tfpi* and *Lims1* significantly increased (Figure 6K). These phenomena significantly improved after applying HEA to the back. The R‐loop detection of the tissue section of this part by IF showed that the R‐loop of the mouse skin tissue increased significantly after UV irradiation along with skin thickening (Figures 6L,M). After HEA treatment, the R‐loop was significantly reduced, and the pathological phenomenon of skin epidermal thickening was alleviated (Figures 6N,O).

The above experimental results showed that the nucleotide analogue HEA can compete with R‐loop for the binding site of COIL, thereby inhibiting the interaction between COIL and R‐loop. The strong UV response was mediated by the R‐loop, which protected the skin from excessive inflammation and injury from UV.

## 3 DISCUSSION

Excessive UV exposure can trigger an inflammatory response in skin cells, and its key regulator is currently thought to be S14G‐human,^34^ CYLD^38^ and others. It is regulated through various signalling pathways, such as MAPK and NF‐kappa B. The HSF family mainly includes HSF1, HSF2 and HSF4, which are involved in cell ageing, stress response, immune response, and cell growth and apoptosis.^39^ Some related small molecular targets, such as LXR, PPAR and GLUT1, can be used as target genes of the HSF family. In‐depth studies have been conducted on HSF1 and inflammation,^40^ but a clear report on the relationship between HSF4 and inflammation and ageing is not yet available. Previous studies have shown that HSF4 is necessary for the growth and differentiation of crystalline bodies during the development of the lens. The study of HSF4 in the skin is relatively limited.

Our study identified for the first time that the transcription factor HSF4 can mediate the response of skin cells to UVR. HSF4 is present in the nucleus and cytoplasm in an inactive and monomeric form and becomes transcriptionally active upon exposure to stress, similar to other HSF family proteins.^41^ Upon activation under stimuli, such as light or heat, HSFs trimerize and translocate to the nucleus, where they bind to the promoter region of HSP genes, induce their activation, and participate in the transcriptional regulation associated with cellular responses to UV stimuli.^42^ HSF4 can interact with the transcriptional cofactor COIL to form a complex under UV stimulation. As the complex can be bound to the R‐loop, the target genes bound by HSF4 are shifted. Transcription of genes, such as *Atg7*, *Tfpi* and *Lims1*, which are related to inflammation and ageing, is initiated. The relationship between COIL and DNA damage is also explained for the first time.

The R‐loop is the product of DNA damage; its transcriptional regulatory role has drawn attention in recent years.^43^ The association of the R‐loop with skin problems has not been reported. Our data showed for the first time that after UV stimulation, many R‐loops are generated in cells, especially in the nucleus. These massively increased R‐loops are believed to be redundant and pathological. UV‐stimulated R‐loop was involved in the transcriptional regulation mediated by the HSF4–COIL complex, and many target genes were involved in the inflammatory response. These results suggested that the formation of a large number of R‐loops is a key step in UV‐induced inflammatory response. Thus R‐loops are potential targets for regulating skin problems. Inhibiting or delaying this step helps improve the adaptability of the skin, thereby improving the skin's oversensitivity to UV rays. On the basis of this idea, the nucleic acid analogues were screened. Both HEA and cordycepin could inhibit a large increase in pathological R‐loops, thereby exerting anti‐inflammatory, anti‐ageing and whitening effects. They may be the first reported potential drugs targeting pathological R‐loops.

As the increase in R‐loops is global, other transcriptional complexes that can bind to R‐loops and play a similar cotranscriptional function cannot be excluded. In addition, a pathological increase in R‐loop can be found in diseases, such as cancer. Future studies will focus on whether the potential drugs discovered targeting pathological R‐loops have broader indications.

## 4 MATERIALS AND METHODS

### 4.1 Animal studies

Animal experiments were conducted in accordance with the National Institutes of Health Animal Use Guidelines. All the experimental protocols were approved by the institutional animal care and use committee at Tianjin International Joint Academy of Biomedicine. The approval code of the ethics committee is 2022‐SYDWLL‐00591. We purchased male C57BL/6 mice (6‐8 weeks old) from Beijing Vitong Lihua. These animals were fed a standard laboratory diet, and a 12 h light/12 h dark cycle was guaranteed. All analyses were double‐blinded and randomized.

Six‐week‐old C57BL/6 male mice with an average body weight of 200 ± 30 g were used for the experiments. The animals were divided into the following groups: a (*n* = 6) control group, b (*n* = 6) exposure to ultraviolet‐B radiation (UV‐B), c (*n* = 6) exposure with UV‐B followed by application of low concentration N6‐(2‐Hydroxyethyl)‐adenosine drug (MedChemExpress) (HEA‐L), and d (*n* = 6) exposure with UV‐B followed by application of high concentration HEA drug (HEA‐H).

### 4.2 UV irradiation

UV‐B lamp model NB‐UVB311 (UV‐B narrow band PL‐L/PL‐S, Philips) with a wavelength of 280–360 nm (peaks 305–310 nm) and a power of 9 J/s was used for two consecutive weeks three times a week. The exposure time to UV‐B radiation was 60 s. Group c,d continued to receive HEA‐L/H treatment for 2 weeks. All groups of mice received euthanasia on the same day. Skin tissues were immersed in paraformaldehyde for 24 h, dehydrated, and embedded in paraffin according to standard procedures. Immunohistochemistry, IF on tissues and HE was performed after sectioning.

### 4.3 IHC staining

After deparaffinization with xylene, rehydration in graded ethanol, immersion in 0.3% hydrogen peroxide, and heat‐mediated antigen retrieval in citric acid (pH 6.0), paraffin sections were obtained from the experiments with a previously described method. The tissue sections were incubated overnight at 4°C with the corresponding antibodies and labelled with horseradish peroxidase secondary antibodies (Affinity) for 60 min at room temperature. Finally, the sections were microscopically observed in DAB solution (Gene Tech) and counterstained with hematoxylin. Expression scoring was performed according to the proportion and intensity of positively stained cells: 0%−5% was scored as 0; 6%−35% scored 1 point; 36%−70% scored 2 points; over 70% scored 3 points. The final score was designated low or high expression as follows: low expression (scores of 0–1) and high expression (scores of 2–3). These scores were independently determined in a blinded fashion by two experienced pathologists and averaged as percentages.

### 4.4 H&E staining

The above‐obtained paraffin section H&E staining kit (Beyotime) was used for evaluation. The normal score of injury area was 0, <25% injury was 1, 25%−50% injury was 2, 50%−75% injury was 3, 75%−90% injury was 4, and >90% injury was 5. At the same time, the thickness of the mouse epidermis was measured.

### 4.5 Cell culture and treatment

NIH‐3T3 cells were purchased from Keygen Biotech. The cells were cultured in DMEM (HyClone) containing 10% fetal bovine serum (FBS) (Thermo Fisher Scientific). And kept at 37°C in an incubator containing 5% carbon dioxide (Thermo Fisher Scientific).

### 4.6 Overexpression and knockout experiments

NIH‐3T3 cells were transfected with Lipo8000 (Beyotime) using HSF4, COIL, Atg7, Tfpi, Lims1, RNaseH overexpression plasmid (Sino‐Biological) or CRISPR knockout plasmid (Santa Cruz) containing Cas9 and guide RNA according to the manufacturer's instructions. Before the selection of purinomycin, the transfected cells were incubated for 48 h (Table S1).

### 4.7 Confocal imaging and analysis

The cells were seeded on glass climbing pieces and cultured at 37°C until they became adherent. After rinsing three times with prechilled phosphate‐buffered saline (PBS) and fixation with prechilled tissue cell fixative (Solarbio), incubation was performed using a blocking punch solution for 2 h. Incubation with the primary antibody was performed at 2 h at room temperature after PBS rinsing, and then incubation with the corresponding secondary antibody was performed for 2 h after PBS rinsing. Slides were finally mounted with DAPI (Solarbio) and imaged using a Zeiss LSM800 confocal microscope.

### 4.8 Duolink in situ PLA

The interaction of HSF4 and COIL was detected using the Duolink kit (Sigma Aldrich) to obtain monoclonal probes for oligodeoxynucleotides. Hybridization between PLA probes results in a fluorescent signal when the distance between the two proteins was ≤40 nm. NIH‐3T3 cells were seeded on glass climbing pieces, rinsed with PBS and fixed using cell tissue fixative (Solarbio) for 10 min. Climbing pieces with cells were blocked using 3% bovine serum albumin (BSA; Solarbio) for 60 min at 37°C after permeabilization in 0.5% Triton X‐100 for 5 min. Cells were then incubated with primary antibodies containing 1% BSA overnight at 4°C, the following day in the dark at 37°C for 60 min with corresponding secondary antibody–conjugated PLA probes. Climbing sections were stained with DAPI (Solarbio) after three washes with buffer. Duolink signals were detected using a Zeiss LSM800 confocal microscope after staining.

### 4.9 Luciferase reporter assay

Luciferase activity was measured using the dual luciferase reporter assay kit (Beyotime) according to the manufacturer's protocol. The fluorescence signal was detected using a multifunctional microplate reader.

### 4.10 Western blot analysis

Cells were washed with PBS and placed on ice and lysed by adding a prechilled protease inhibitor cocktail (Solarbio) for 30 min. An appropriate amount of SDS loading buffer was added to the protein samples and placed in a metal bath at 95°C to heat for 10 min. Protein products were separated by electrophoresis and transferred to polyvinylidene difluoride (PVDF) membranes and incubated overnight at 4°C with primary antibodies against HSF4 (Affinity), COIL, (Proteintech), LIMS1 (Affinity), ATG7 (Affinity), TFPI (Affinity), H2AX (Zenbio) and GAPDH (Affinity) and then for 1 h at room temperature in horseradish peroxidase conjugated goat anti‐rabbit or goat anti‐mouse IgG secondary antibodies (Beyotime). Detection was performed using an enhanced chemiluminescence kit (Vazyme).

### 4.11 Pulldown

Proteins were first extracted for Western blot with a pull‐down kit (Thermo Fisher Scientific) according to the product requirements. The antibody that targets proteins or the antibody cognate IgG (negative control) was coupled to activated A/G beads, and the antibody‐coupled beads were incubated with proteins overnight at 4°C. After the proteins were eluted from the beads, the proteins were separated using a 10% SDS‐PAGE gel and transferred to a PVDF membrane (Millipore) for Western blot analysis.

### 4.12 Co‐IP assay

After rinsing three times with ice‐cold PBS, IP lysis buffer (Solarbio) was added, and the total protein in the lysis solution was used as an “input” sample. Protein A/G beads were incubated with specific antibodies or IgG for 6–8 h, and the formed immune complex was incubated with cell lysate overnight. Then the proteins precipitated from the beads were removed. Finally, the products of immunoprecipitation or cell lysate products were separated by 10% SDS‐PAGE gel and transferred to the PVDF membrane for Western blot analysis.

### 4.13 CUT&RUN

In this experiment, a CUT&RUN kit was used for extraction (CST). An appropriate number of living cells were prepared. First, concanavalin A beads were combined with the target primary antibody HSF4 and COIL successively. After binding overnight, the beads were eluted with PAG mnase enzyme to digest and release the eluted DNA. Finally, a DNA purification kit (CST) was used to purify the obtained DNA fragment bound to the target protein. The obtained DNA was stored in a DNA evolution buffer (Cell Signaling).

### 4.14 Chromatin immunoprecipitation

ChIP assays are performed using the Pierce Agarose ChIP (Thermo Scientific #26156) kit to allow protein–DNA complexes to remain stable, followed by extraction. In‐vivo cross‐linking is usually achieved using formaldehyde. Cross‐linking directly in a cell locks in the protein–DNA complex, thereby trapping these unstable and sometimes transient interactions. In the lysis, extraction, and solubilization of the cross‐linked complexes, the ChIP assay kit included the Thermo Scientific Chromatin Preparation Module. This method can be used to isolate chromatin‐bound DNA and enrich samples for target proteins without the use of a Doens homogenizer and with less than 15% contamination from other cell compartments.

### 4.15 qPCR and ChIP qPCR

Quantitative PCR was performed as described previously. The sequences of the primers used are provided in Table S2.

### 4.16 Chromatin immunoprecipitation–reChIP

ChIP–reChIP refers to the process of immunoprecipitation of another target protein without uncoupling on the basis of the first ChIP to obtain DNA sequences that bind to HSF4 and COIL target proteins. Notably, the amount of DNA enriched by two times of immunoprecipitation is relatively small, so we concentrated the DNA through multiple times of immunoprecipitation before operation during analysis.

### 4.17 DNA–RNA immunoprecipitation

DRIP was performed as described by Hamperl et al.^44^ DNeasy Blood & Tissue Kits (QIAGEN) was used to prepare the total nucleic acid of cells and S9.6 antibody immunoprecipitate DNA–RNA structure from total nucleic acid according to the requirements of the kit. DNA–RNA complex was purified and analyzed as a ChIP sample.

### 4.18 CUT&RUN data analysis

First, according to the sequencing data fed back by the sequencing company, we used ctrl‐hsf4, Ctrl coil, uv‐hsf4, and uv‐coil.BW file for data analysis. H3k4me3 sequencing data were obtained from Cistrome. Next, we used IGV to align the readings with the mouse genome (mm10). For DRIP‐seq, we referred to the GSM1720621.Bigwig file for analysis. The total reading was standardized. Owing to the advantages of the high signal‐to‐noise ratio of CUT&RUN technology, even in the experimental data with low cell size, its signal‐to‐noise ratio was higher than the results of the ChIP samples.

### 4.19 RNA‐sequencing

The cells collected by centrifugation were rapidly dissolved in TRIzol for lysis, with a reference dosage of 1 ml of TRIzol per 5 × 10^6^ cells. They were allowed to stand at room temperature for 5 min before they were transferred for low‐temperature storage at −80°C. Nanodrop2000 (Thermofish) was used for concentration detection of the extracted nucleic acids, and Agent2100, LabChip GX for integrity testing. Place mRNA Capture Beads on a four‐dimensional rotator and mix thoroughly. Balancing was performed 30 min before use. After combining mRNA with magnetic beads twice, the above mRNA products were added to the PCR instrument, and the corresponding interruption conditions were determined based on the sample quality and other conditions. After mixing and centrifugation, the first strand of cDNA was synthesized using a program set in the PCR instrument. The second strand of cDNA was synthesized, and the second strand was purified. The final reagent was added to the purified product mentioned above and placed in the PCR instrument for reaction, and the connector was immediately connected after the reaction was completed. The connectors and ligases were added to the above reaction products successively and incubated in a metal bath, and connector connection reactions were conducted. After the connecting product was purified, segment selection was performed, followed by PCR amplification and purification. Finally, the library was subjected to quality inspection with the Qsep‐400 method.

Illumina novaseq6000 was used for the machine sequencing of the constructed library. The PE150 sequencing platform was Illumina NovaSeq 6000 platform (San Diego).

### 4.20 Generation of 3D skin organoid

Prepare neutralized type I collagen on ice, following the manufacturer's recommendations. As the final concentration, use 3 mg/ml for type I collagen (stock concentration of type I collagen is 3.47 mg/ml), and make sure the final volume of the mixture is 5 ml. Calculate the volume of 10× PBS (final volume/10 = 0.5 ml). Calculate the volume of type I collagen to be used (final volume × final collagen concentration/stock collagen concentration = 5 ml × 3 mg/ml/3.47 mg/ml = 4.32 ml). Calculate the volume of 1 N NaOH (volume of collagen to be used × 0.023 ml = 0.1 ml). Calculate the volume of dH_2_O (final volume—volume of collagen—volume of 10× PBS—volume of 1 N NaOH = 5 ml ‐ 4.32 ml ‐ 0.5 ml ‐ 0.1 ml = 0.08 ml). Mix the contents of the tube and keep it on ice until ready to use. Add 1 ml of EDTA to the iPSC‐derived fibroblasts and incubate at 37°C with 5% CO_2_ for 2 min. Harvest the detached cells, count the cells using a hemocytometer, and transfer 2 × 10^5^ cells to a new 15 ml conical tube. Centrifuge at 250 × *g* for 2 min and remove the supernatant. Resuspend the cells of the iPSC‐derived fibroblasts in 1.5 ml of FDM1 and neutralized the type I collagen solution (1:1). Place the membrane insert on a 6‐well microplate, transfer the mixture to the insert, and incubate at RT for 30 min. After confirming the gelation, add 2 ml of medium to the top of the insert and 3 ml to the bottom of the well. Incubate the matrix of fibroblasts and collagen at 37°C with 5% CO_2_ for 5–7 days, until the gelation is complete and no longer contracts.After the complete gelation, detach the iPSC‐derived keratinocytes using EDTA. Add 1 ml of EDTA and incubate at 37°C with 5% CO_2_ for 2 min. Harvest the detached cells, count them using a hemocytometer, and transfer 1 × 10^6^ cells to a new 15 ml conical tube. Centrifuge at 250 × g for 2 min.

Remove the supernatant and resuspend 1 × 10^6^ cells in 50−100 μl of low calcium epithelial medium 1 (EP1). Aspirate all medium in the matrix and seed 1 × 10^6^ cells of the iPSC‐derived keratinocytes onto each fibroblast layer. Incubate the plate at 37°C with 5% CO_2_ for 30 min. Add 2 ml of EP1 to the top of the insert and 3 ml of EP1 to the bottom of the well. After 2 days, aspirate all medium in the membrane insert plate and change the medium to normal calcium EP2 for 2 days. After 2 days, aspirate all medium and add 3 ml of the cornification medium only to the bottom to generate an air‐liquid interface. Maintain the 3D skin organoid for up to 14 days at 37°C with 5% CO_2_ and change the medium every other day. Harvest the 3D skin organoid by cutting the edge of the insert, and use it for a further study of staining and skin graft.

### 4.21 Statistical analysis

Statistical analysis was performed using GraphPad Prism (version 8) for all other experiments as described in the sections. Unless otherwise stated, means and standard deviations from three or more independent experiments were plotted.

## AUTHOR CONTRIBUTIONS

Conceptualization: Heng Zhang, Tao Sun and Yi‐qian Feng; Methodology: Yi‐qian Feng, Heng Zhang, Lu‐ning Qin, Lei Zhang and Qing‐qing Li; Investigation: Heng Zhang, Yi‐qian Feng and Lei Zhang; Visualization: Yi‐qian Feng, Jing‐xia Han and Lei Zhang; Funding acquisition: Hui‐juan Liu and Tao Sun; Project administration: Yi‐qian Feng and Heng Zhang; Supervision: Hui‐juan Liu and Tao Sun; Writing—original draft: Yi‐qian Feng and Heng Zhang; Writing—review & editing: Yi‐qian Feng and Heng Zhang

## CONFLICT OF INTEREST STATEMENT

The authors declare no conflict of interest.

## FUNDING INFORMATION

This work was supported by grants from the National Natural Science Foundation of China (grant nos. 82073205, 82272934 and 82273237); the National Youth Talent Support Program (2020); the Natural Science Foundation of Tianjin (19JCJQJC63200 and 21JCZDJC00930); and the Fundamental Research Funds for the Central Universities (63233164). China Postdoctoral Science Foundation (2021M701778).

## Supporting information

Supporting Information

Supporting Information

Supporting Information

## Data Availability

CUT&RUN sequencing has a higher signal‐to‐noise ratio than chromatin immunoprecipitation (ChIP) sequencing results and can be analyzed simultaneously with ChIP data in the database, and we have made the genes obtained from sequencing available as GEO (GSE233680) for readers to review.
