# Supplementary material for: HSF4/COIL complex‐dependent R‐loop mediates ultraviolet‐induced inflammatory skin injury
Source: Clin Transl Med. 2023 Jul 17;13(7):e1336. doi: 10.1002/ctm2.1336 (PMC10352565; doi:10.1002/ctm2.1336)
Supplement: Supplementary file 1 — Supporting Information [file CTM2-13-e1336-s002.docx]

Supplementary Materials for

HSF4/COIL complex-dependent R-loop mediates UV-induced inflammatory skin injury

**Authors:** Yi-qian Feng^1#^，Heng Zhang^1,2#^ ,Jing-xia Han^1,2^，Bi-jia Cui^1^，Lu-ning Qin^1^，Lei Zhang^1^，Qing-qing Li^1^，Xin-ying Wu^1^，Nan-nan Xiao^1^，Yan Zhang^1^，Ting-ting Lin^3^* ，Hui-juan Liu^1,2^*，Tao Sun^1^*

**Affiliations:**

1，State Key Laboratory of Medicinal Chemical Biology and College of Pharmacy, Nankai University, Tianjin, China;

2，Tianjin International Joint Academy of Biomedicine, Tianjin, China;

3，Medical plastic and cosmetic center，Tianjin Branch of National Clinical Research Center for Ocular Disease, Tianjin Medical University Eye Hospital ,Tianjin, China

^#^Contributed equally

Correspondence: Tao Sun*, email: sunrockmia@hotmail.com

Hui-juan Liu*, email: [liuhuijuanxyz@163.com](mailto:liuhuijuanxyz@163.com)

Ting-ting Lin^2^*,email：ltt6123@126.com

**This PDF file includes:**

CUT&RUN method

Figures. S1

Figures. S2

Tables S1

Tables S2

**CUT&RUN method**

(1) The addition of data analysis is as follows: first, the fastq file of the analysis target was obtained, and quality control analysis of the data was performed by removing the connectors (fastp) and checking the data quality (fastqc). hisat2 compares the target file with the genomic file to obtain the sam file, and then converts it into a bam file by using samtools. The next step of analysis was then performed. The main software programs used are fastp, fastqc, hisat2, samtools. Ilumina, and other second-generation sequencing methods. Data containing connectors were removed with fastp before quality control analysis. After removing the junction, the sequencing quality was checked and assessed using FastQC (v0.11.4).

Our data passed the sequence sequencing quality statistics; as such, the GC content distribution was checked without AT and GC separation phenomenon.

(2) With regard to the read alignment, we uploaded the bam format file with the gene comparison results. We selected File -> Load From File and imported the bam file for analysis after selecting the sequencing of each sample.

(3) Only the reads that were compared to the unique position of the reference genome during the analysis were used for peak identification. In theory, reads tend to be enriched at and near the protein–DNA interactions. Peak calling was used to identify unique mapped reads on the genome.

By identifying the enriched regions on the genome through peak calling, we can obtain information on potential protein–DNA interactions.

We used MACS2 (Yong Zhang, Tao Liu et al., 2008) software for peak extraction and Poisson distribution model (p-value criterion of 1e-5) to examine the whole genome. MACS2 scans the genome with a certain window size (300 bp), counts the enrichment of reads in each window, and then uniformly analyzes the enrichment of reads. We take a sample of the windows evenly to build a “bimodal model” (the figure below). Finally, the distance between the two peaks was considered as the length d of the binding site, and each read was extended to d/2 of the length left and right. When the number of reads in a certain interval is screened for significant enrichment, it is defined as a potential peak.

(4) We annotated peaks using the R package ChIPseeker (Yu G, Wang L, He Q, 2015). The information of peak annotation (intercepted part of data display) was presented.


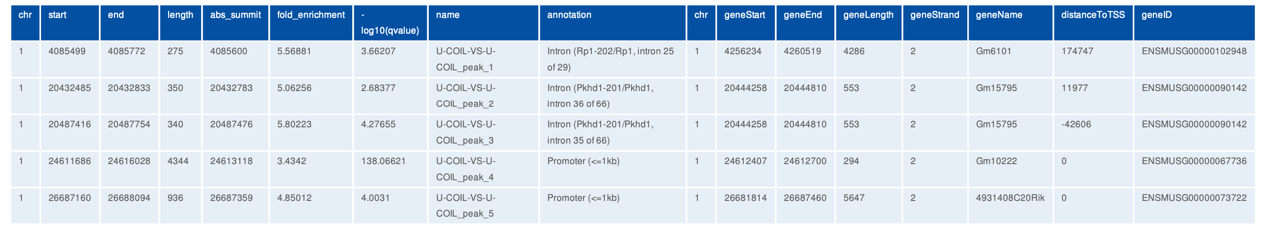


(5) Finally, differential peaks were analyzed.

The location of peaks in the genome was determined by their position in the genome: promoters, 5-UTRs, 3-UTRs, exons, introns, downstream, intergenic. The priority settings were as follows: eukaryotic for Promoters>5-UTRs>3- UTRs>Exons>Introns> Downstream>Intergenic; Protocore is Promoters>5-UTRs>3-UTRs>gene>Downstream>Intergenic. The percentage of peaks in each region to the total peaks was counted.

The peaks from the two experimental groups were merged using bedtools (version, 2.25.0). The reads counts of the merged peak regions were counted. Differential analysis was performed using edgeR (V3.24.1) of the Bioconductor package. Differential peaks were screened based on the test results according to different significance criteria (more than twofold change in the read counts of differential peaks and qvalue (fdr, padj) <= 0.05). The significant difference up and down of peaks were counted.

**Figure S1** **Expression of inflammatory aging factors in human organs**
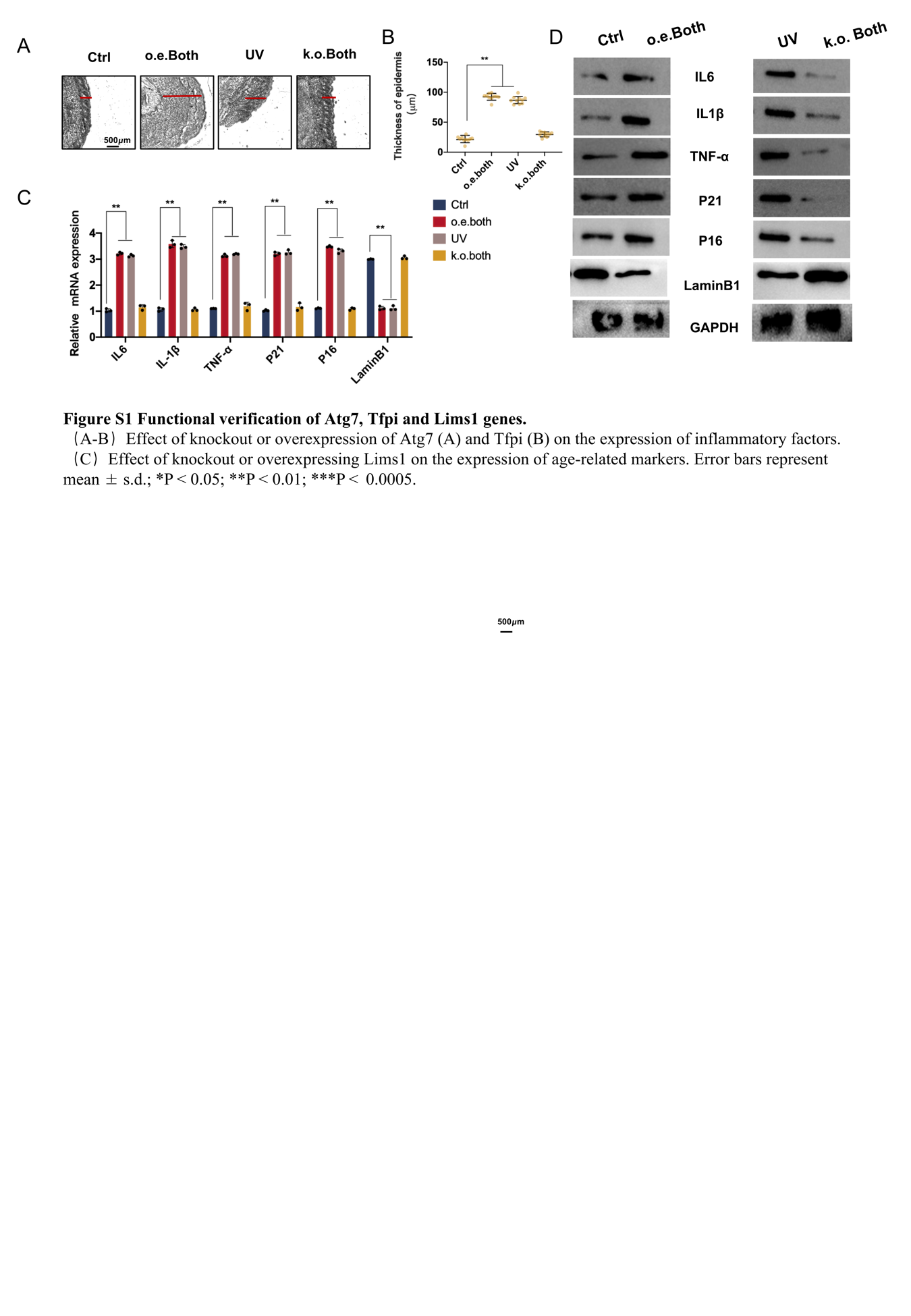


（A）Constructed human skin like organs. Among them, o.e.both refers to the simultaneous expression of HSF4 and COIL genes, while k.o.both refers to the simultaneous knockout of HSF4 and COIL genes.

（B）According to the statistics of skin thickness of human skin like organs that o.e. HSF4

and COIL can make organoid cutin accumulate, and there is similarity between epidermal thickening and UV-induced injury model. k.o. HSF4 and COIL can reduce the epidermal thickness of organoids.

（C）The qPCR data showed that the expression of inflammation and aging related mRNA in human skin organs increased under ultraviolet stimulation, while the expression of inflammation and aging related mRNA increased after o.e.HSF4 and COIL. The same result existed after ultraviolet induction, while k.o. HSF4 and COIL could alleviate the expression of inflammation and aging related mRNA.

（D）The Western blot data showed that the expression of inflammation and aging related。protein in human skin organs increased under UV stimulation, while the expression of inflammation and aging related protein increased after o.e.HSF4 and COIL. The same result existed after UV induction, while k.o. HSF4 and COIL could alleviate the expression of inflammation and aging related protein.

**Figure S2 Validation of knockout/overexpression cell lines**


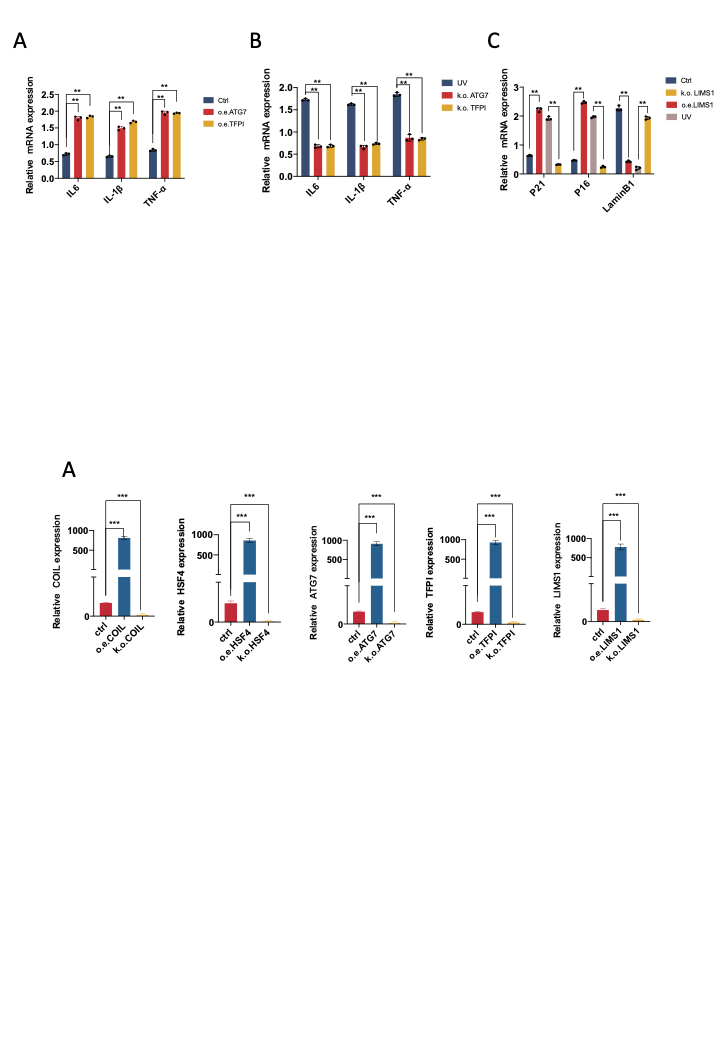


（A）The QPCR experimental data showed mRNA expression levels after knocking out or overexpressing genes in NIH-3t3.

**Table S1.**

**Table 1. gene knockout sequences**

| **gRNA name** | **gene target** | **sequence** |  |
| --- | --- | --- | --- |
| HSF4-gRNA1  HSF4-gRNA2  HSF4-gRNA3  COIL-gRNA1  COIL-gRNA2  COIL-gRNA3  ATG7-gRNA1  ATG7-gRNA2  ATG7-gRNA3  TFPI-gRNA1  TFPI-gRNA2  TFPI-gRNA3  LIMS1-gRNA1  LIMS1-gRNA2  LIMS1-gRNA3  Rnh1- gRNA1  Rnh 1-gRNA2  Rnh 1-gRNA3 | HSF4  HSF4  HSF4  COIL  COIL  COIL  ATG7  ATG7  ATG7  TFPI  TFPI  TFPI  LIMS1  LIMS1  LIMS1  Rnh1  Rnh1  Rnh1 | CTGTCATCGCCTCGCAGCGC  CGATGACAGTCGATGGCGTC  CAGCATCCGAGCTTCGTGCG  TTTGTCCGCCCCGTCTCATC  GAAGCCTCGGTAAAGCCCGA  AGAGGTCCTCTCCTCGCTAC  GAAGTTGAACGAGTACCGCC  ATTTACTTACCGCGAAGGTC  AAAGGGGGCGAACTGCAACT  CAGTGTGAACGATTCGTGTA  CGAGAACCGATTTGATACCC  GAGGACCCTGGACTCTGCCG  CACATACGAAGCACTGTTCG  CGCAGTTGGCACAGTTGAAG  TGCCACGTGTGAGCGCTGCA  GGCCTCCAAAGCTTCGCTAC  CAGTTCCTGTAGCGAAGCTT  TCTTACAAGTGGGATTCTGC |  |

**Table S2.**

**Table 2. Primers of PCR**

| **Gene name** | **Application** | **sequence** |  |
| --- | --- | --- | --- |
| ATG7-F  ATG7-R  Atg7-F  Atg7-R  Atg7-F  Atg7-R  Atg7(R1)- F  Atg7(R1)- R  Atg7(R2)- F  Atg7(R2)- R  Atg7(R3)- F  Atg7(R3)- R  TFPI-F  TFPI-R  Tfpi-F  Tfpi-R  Tfpi-F  Tfpi-R  Tfpi(R1)- F  Tfpi(R1)- R  Tfpi(R2)- F  Tfpi(R2)- R  Tfpi(R3)- F  Tfpi(R3)- R  LIMS1-F  LIMS1-R  Lims1-F  Lims1-R  Lims1-F  Lims1-R  Lims1(R1)- F  Lims1(R1)- R  Lims1(R2)- F  Lims1(R2)- R  Lims1(R3)- F  Lims1(R3)- R  IL‑6-F  IL‑6-R  IL-1β-F  IL-1β-R  TNF-α-F  TNF-α-R | RT-qPCR  RT-qPCR  RT-qPCR  RT-qPCR  ChIP-qPCR  ChIP-qPCR  ChIP-qPCR  ChIP-qPCR  ChIP-qPCR  ChIP-qPCR  ChIP-qPCR  ChIP-qPCR  RT-qPCR  RT-qPCR  RT-qPCR  RT-qPCR  ChIP-qPCR  ChIP-qPCR  ChIP-qPCR  ChIP-qPCR  ChIP-qPCR  ChIP-qPCR  ChIP-qPCR  ChIP-qPCR  RT-qPCR  RT-qPCR  RT-qPCR  RT-qPCR  ChIP-qPCR  ChIP-qPCR  ChIP-qPCR  ChIP-qPCR  ChIP-qPCR  ChIP-qPCR  ChIP-qPCR  ChIP-qPCR  RT-qPCR  RT-qPCR  RT-qPCR  RT-qPCR  RT-qPCR  RT-qPCR | CGTTGCCCACAGCATCATCTTC  CACTGAGGTTCACCATCCTTGG  CCTGTGAGCTTGGATCAAAGGC  GAGCAAGGAGACCAGAACAGTG  GAAGTTGAGCGGCGGTAAGTAAG  CTTTAAGGGGATGCAGAACCTCT  CCGCTGGCATTTCTGTTGCTGT  CAGGGATCACCTCCAAATTAGCG  GGGAAGCAAGTTTGTCTGGACC  AAACTGCTCCGCTGAAGACTGG  CCTAGTGATAAGGAATGCACGATG  CTAGGCAGGTTTGATCTCCGTTC  TGTGAAGGCAGCATCTGGAGCA  CCGTACACGAATCGTTCACACTG  CATGAGATGAGAAGGGAAGGTAC  CTAGAAATTGCAAAGTATATCAGAA  TGCGAGGGGATGCAGTAAAG  GCCTCCAAACTTCCTCTGCT  ACTAAAGTCACACAGTGCTGAG  TGAAGACCACTGCCCAAGAG  CTGAGAGGTACGGAAGCTGC  CCTAACAGTGGAGCATGCCA  GCTCAGGGAAGACTGCTGAG  GGCGTCGTGAGTTCTTTCCT  GGGTTTGTCAAGAATGCTGGCAG  GCACAGTTGAAGTGGTCTGGATG  TCCAGAGCTTGAAGGTGTTGCC  AACCAAGGGAGCTTCAGGGTCA  CTCTCAGAAAACTTCGCCCC CGTACCTGTTGGTCATTCCG  AACTTCCGGGTTCAAGCAAC  AAGTTCTCTTCGCTGGTGCA  TGTGCACCTAGGGTCTTGTG  CGCTGTCTGTCTGTTGCTTC  GGGGAAGTGAAGCCCTCTTC  TGGTATTGCAGGTGGAGCAG  TGCAGTTCCAGCTTCGATACCG  CTGCAAGTGCATCATCGTTGTTC  TGGACCTTCCAGGATGAGGACA  GTTCATCTCGGAGCCTGTAGTG  CTCTTCTGCCTGCTGCACTTTG  ATGGGCTACAGGCTTGTCACTC |  |

[1] FastQC: http://www.bioinformatics.babraham.ac.uk/projects/fastqc/

[2] Cock, P.J.A., Fields, C.J., Goto, N., Heuer, M.L., and Rice, P.M. The Sanger FASTQ file format for sequences with quality scores, and the Solexa/Illumina FASTQ variants. Nucleic acids research 38, 1767-1771

[3] Heinz S, Benner C, Spann N, Bertolino E et al. Simple Combinations of Lineage-Determining Transcription Factors Prime cis-Regulatory Elements Required for Macrophage and B Cell Identities. Mol Cell 2010 May 28;38(4):576-589.

[4] Yan H, et al. HiChIP: a high-throughput pipeline for integrative analysis of ChIP-Seq data.BMC Bioinformatics. 2014 Aug 15;15:280. doi: 10.1186/1471-2105-15-280.

[5] Mendoza-Parra MA, et al. A quality control system for profiles obtained by ChIP sequencing. Nucleic Acids Res. 2013 Nov;41(21):e196. doi: 10.1093/nar/gkt829.Epub 2013 Sep 14.

[6] Lelandais G, et al. ChIPseq in Yeast Species: From Chromatin Immunoprecipitation to High-Throughput Sequencing and Bioinformatics Data Analyses. Methods Mol Biol. 2016;1361:185-202. doi: 10.1007/978-1-4939-3079-1_11.

[7] Zhang Y, Liu T, Meyer C A, et al. Model-based analysis of ChIP-Seq (MACS)[J]. Genome biology, 2008, 9(9): R137.

[8] Yu G, Wang L G, He Q Y. ChIPseeker: an R-Bioconductor package for ChIP peak annotation, comparison and visualization[J]. Bioinformatics, 2015, 31(14): 2382-2383.

[9] Bardet A F, He Q, Zeitlinger J, et al. A computational pipeline for comparative ChIP-seq analyses[J]. Nature protocols, 2012, 7(1): 45.

[10] Landt S G, Marinov G K, Kundaje A, et al. ChIP-seq guidelines and practices of the ENCODE and modENCODE consortia[J]. Genome research, 2012, 22(9): 1813-1831.

[11] Steube A, Schenk T, Tretyakov A, et al. High-intensity UV laser ChIP-seq for the study of protein-DNA interactions in living cells[J]. Nature communications,2017, 8(1): 130

[12] Ma W, Noble W S, Bailey T L. Motif-based analysis of large nucleotide data sets using MEME-ChIP[J]. Nature protocols, 2014, 9(6): 1428.

[13] Gene Ontology Consortium. (2004). The Gene Ontology (GO) database and informatics resource. Nucleic acids research, 32(suppl_1), D258-D261. (GO)

[14] Kanehisa, M., & Goto, S. (2000). KEGG: Kyoto encyclopedia of genes and genomes. Nucleic acids research, 28(1), 27-30. (KEGG)
